# A Method of Neighbor Classes Based SVM Classification for Optical Printed Chinese Character Recognition

**DOI:** 10.1371/journal.pone.0057928

**Published:** 2013-03-11

**Authors:** Jie Zhang, Xiaohong Wu, Yanmei Yu, Daisheng Luo

**Affiliations:** Image Information Institute, School of Electronics and Information Engineering, Sichuan University, Chengdu, China; University of Adelaide, Australia

## Abstract

In optical printed Chinese character recognition (OPCCR), many classifiers have been proposed for the recognition. Among the classifiers, support vector machine (SVM) might be the best classifier. However, SVM is a classifier for two classes. When it is used for multi-classes in OPCCR, its computation is time-consuming. Thus, we propose a neighbor classes based SVM (NC-SVM) to reduce the computation consumption of SVM. Experiments of NC-SVM classification for OPCCR have been done. The results of the experiments have shown that the NC-SVM we proposed can effectively reduce the computation time in OPCCR.

## Introduction

For Chinese characters, there are hundreds of similar character groups, such as 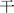 and 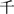, 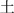 and 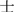, 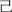, 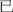, and 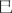, and so on. In each group, it is difficult to classify the similar characters in optical printed Chinese character recognition (OPCCR). To overcome the difficulty, many classifiers [1]–[9] have been developed. Some experiments [4] [10]–[13] in recent years showed that SVM method can achieve high identification rate (IR) in many dataset, such as MNIST dataset, ETL8B dataset, ETL9B dataset, etc. However, SVM [1]–[4] is a classifier used for two classes. When it is utilized for large multi classes in OPCCR, its computation is time-consuming. This is because that the training and classification of multi classes in SVM have to be performed between each pair of the all classes. For this reason, several methods have been proposed to reduce the computation of SVM.

A typical method, *k* nearest neighbors SVM (SVM-KNN) [5]–[6], was proposed to reduce the number of training procedures in SVM. In the SVM-KNN method [6], first, the *k* nearest neighbors (KNN) of a query sample are found by calculating the distances of the query to all the training samples. Then to the *k* nearest neighbors, if the labels of these samples are different, the pairwise distance matrix between the *k* neighbors is computed, and converted into a kernel matrix, and then directed acyclic graph SVM (DAGSVM ) [14] is trained by the kernel matrix. Although the training procedures of SVM are reduced, it leads to the additional calculation time of the *k* nearest neighbors to all the training examples. Although a local nearest neighbor (LNN) classifier method was proposed in a combined SVM and KNN method [7], it was used to improve SVM, but not to reduce the computation. The local nearest neighbor samples were extracted to construct local SVM classifiers when global SVM classifying failed. The class boundary provided by local SVM is utilized to learn a query-dependent boundary-driven metric where *k* nearest neighbors are found, followed by KNN classifier to the query. Similarly, a nearest neighbor classifier (NNC) method was proposed with SVM in [8], when the outputs of global SVM classifiers are different labels. It was proposed to increase the classification of SVM, not to reduce the computation consumption of recognition. The global SVM method is applied in the training step. In the test process, both global SVM and NNC are utilized to classify the query sample. Another typical method, local adaptive SVM classifier (LASVM) [9], was proposed. In training procedure, a LASVM classifier is trained according to the date-dependent distance matrix of training data. In classification procedure, if the *k* nearest neighbors of the query sample have more than one label, the local SVM is utilized for further recognition. The *k* nearest neighbors have commonly different labels, so the LASVM method is less effective to computation reduction. To reduce further the computation of SVM, we propose here a method of NC-SVM for OPCCR. In our method, first, the pattern space of Chinese characters is partitioned into subspaces. Corresponding to a subspace, a container is constructed. The classes in the subspace are stored in the corresponding container. Then, in the training procedure of OPCCR, a known training sample and the neighbor classes (NCs) of the sample are used to train SVM. In the classification procedure, an unknown sample is classified by SVM using NCs of the unknown sample. Thus, the computation of OPCCR by SVM is reduced greatly. To illustrate the method of NC-SVM and compare it with other local neighbor based SVM methods, firstly, the pattern space partitioning is introduced. Next, the training of SVM and NC-SVM and the classification by SVM and NC-SVM are stated. Then, the performance of NC-SVM in OPCCR is represented. Last, some experimental results of OPCCR by NC-SVM are given. Meanwhile, the computation consumption of NC-SVM is compared with SVM-KNN [6], GsvmLBDknn [7], SVM-NNC [8], and LASVM [9], and modified quadratic discriminant function 2 (MQDF2) [15].

## Methods

### 2.1 Pattern Space Partitioning of NC-SVM

**Figure 1:**
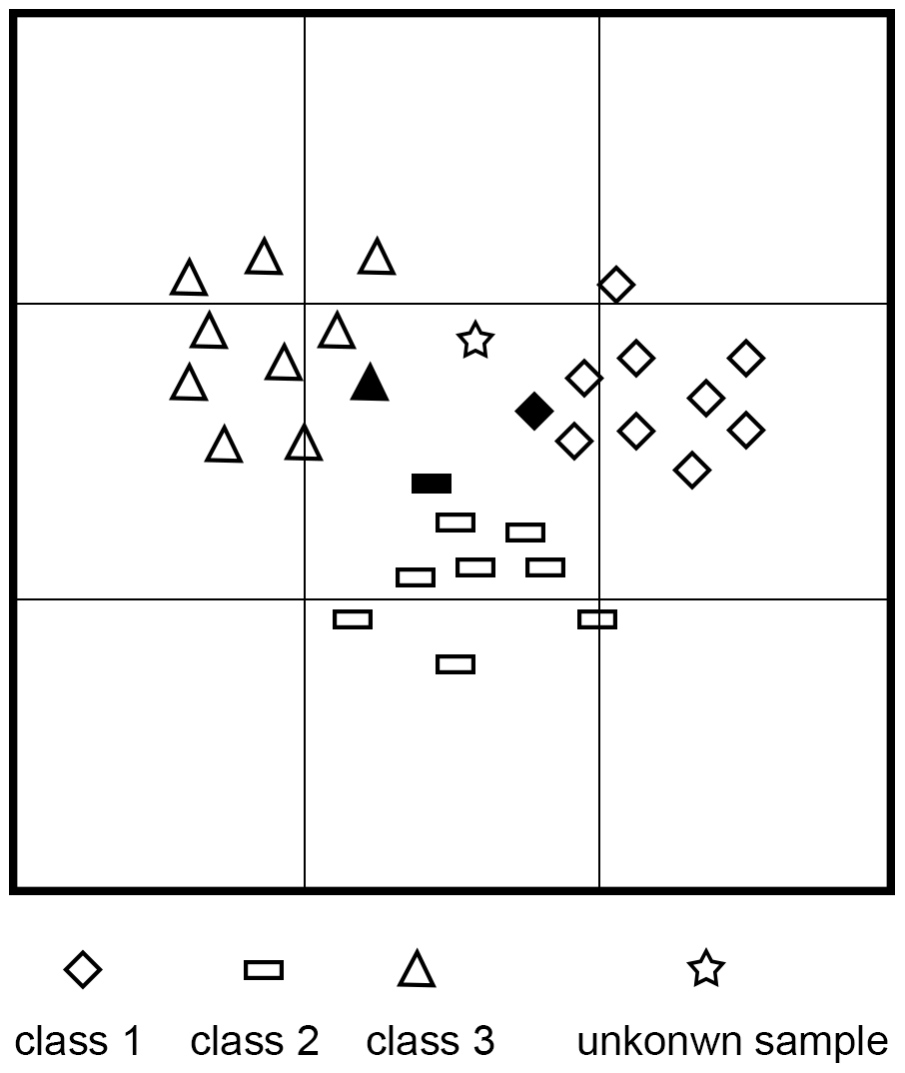
Two dimensional pattern space partitioning. The pattern space of two dimensions is divided into 3^2^ subspaces. The subspace {*u_01_, u_11_*} in the center corresponds to container c_4_. The number of the container is calculated by Equation (6). The NCs in this subspace, i.e. class 1, 2, and 3, are saved in container c_4_ = {1,2,3}.

As we know, SVM algorithm is suitable for the classification of two linearly or nonlinearly separable classes. However, for the classification of multi-classes by SVM, every two classes of all the classes have to be involved in the classification of an unknown sample. In fact, it is not necessary to use all of the classes in OPCCR by SVM. It is because that a class nears to only some but not all of the classes. Hence, to classify an unknown sample, only its neighbor classes (NCs) are used. The problem is how to determine which neighbor classes are used. To solve this problem, we partition the pattern space of Chinese characters into subspaces. In a subspace, the classes falling into this subspace are thought to be neighbors. Thus, when an unknown sample is classified, the classes in the subspace where the unknown sample stands are used by SVM. An example of two dimensional pattern space partitioning and an unknown sample is shown in Figure 1. In the figure, the 2D pattern space is partitioned into 9 subspaces. When an unknown sample stands in the central subspace, the classes in the central subspace: class 1, 2, 3 are its neighbor classes.

Let *P^D^* be a *D* dimensional pattern space of optical printed Chinese characters (OPCCs):
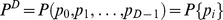
(1)Where 0≤*p_i_*≤1 is the *i*th feature, *i* = 0, 1, …, *D*−1. Each dimension of the pattern space is divided into *U* units.
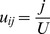
(2)


The unit number *j* can be calculated by:
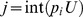
(3)


Thus, the pattern space *P^D^* is partitioned into *U^D^* subspaces:
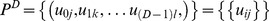
(4)

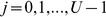
(5)Corresponding to a subspace {*u_ij_*}, a container *c_m_* is constructed. In the container *c_m_*, the numbers of the classes in the subspace {*u_ij_*} are stored. The number *m* is calculated by:
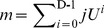
(6)


For example, in Figure 1, *D* = 2, *U* = 3. Corresponding to the subspace {*u_01_, u_11_*}, the unit number of two dimension is (1, 1), *m* = 1×1+1×3 = 4.

### 2.2 Training of SVM and NC-SVM

In the case of multi classes, for every two classes *a* and *b*, one super plane.
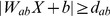
(7)is to be generated to separate the two classes *a* and *b*. Here *a*, *b* = 1, 2, …, *L*, *L* is the number of the classes. Hence, for class *a*, the number *n_a_* of the super planes should be generated is given by:



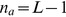
(8)For all the classes, the number *n_t_* of the super planes is
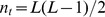
(9)


In each super plane, the matrix *W_ab_* has to be optimized using the training samples *X_a_* selected from class *a*, and *X_b_* from class *b* to satisfy the super plane. Obviously, for a class, when one training sample is used, then (*L*−1) times of training are needed to generate (*L*−1) super planes. For all the classes, *L*(*L*−1)/2 times training are needed. When *S* training samples are used, for a class, the times *t_a_* of training are needed:
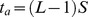
(10)


For all the classes, the times *t_t_* of training are needed:
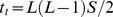
(11)


Hence, the training of SVM is time-consuming.

In NC-SVM, if a training sample has *L_t_* neighbor classes, then the number of the super planes needed to be generated is.
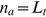
(12)


For example as in Figure 1, the bald training sample of class 2 has two neighbor classes: class 1 and class 3. When it is used, only two super planes of class 2 and 1, class 2 and 3 are need to be trained. Similarly, the bald training sample of class 3 has two neighbor classes: class 1 and class 2. When it is used, only two super planes of class 3 and 1, class 3 and 2 are need to be trained. Thus the total of the super planes to be trained is 3. When *S* training samples are used, the times of training are needed:
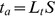
(13)


Thus, for all the classes, the times of training are needed:
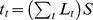
(14)


There are about *L* = 6,000 commonly used Chinese characters. A group of similar characters has not more than *L_t_* = 10 characters. In OPCCR, when the pattern space is partitioned uniformly into more than 6,000 subspaces, the number of NCs *L_t_* is smaller than *L*. Comparing Equations (14) with Equation (11), the training time of NC-SVM is much less than that of SVM.

### 2.3 Classification by SVM and NC-SVM

In the case of multi classes, if starting up from class a, the super planes of class *a* and each of the other classes *b* are used to classify the sample *X*. If the sample *X* satisfies the super plane:
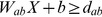
(15)


Then *X* is classified into class *a*. Otherwise:
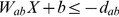
(16)


Then *X* is classified into class *b*. Thus, for *L* classes, the number of the super planes needed to classify *X* is:
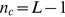
(17)


Hence, the classification of SVM is time-consuming.

NC-SVM can reduce the classification time. In NC-SVM if an unknown sample has *L_u_* neighbor classes, the number of the super planes needed to classify the unknown sample *X* is:
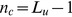
(18)


For example, in Figure 1, the unknown sample has 3 neighbor classes. When starting up from class 1, 2 super planes of class 1 and 2, class 1 and 3 or 2 super planes of class 1 and 2, class 2 and 3 are needed to classify the unknown sample. In OPCCR, most of *L_u_* is smaller than *L*. Hence, comparing Equation (18) with Equation (17), the classification time of NC-SVM is much less than that of SVM.

### 2.4 Implementation of NC-SVM in OPCCR

**Figure 2:**
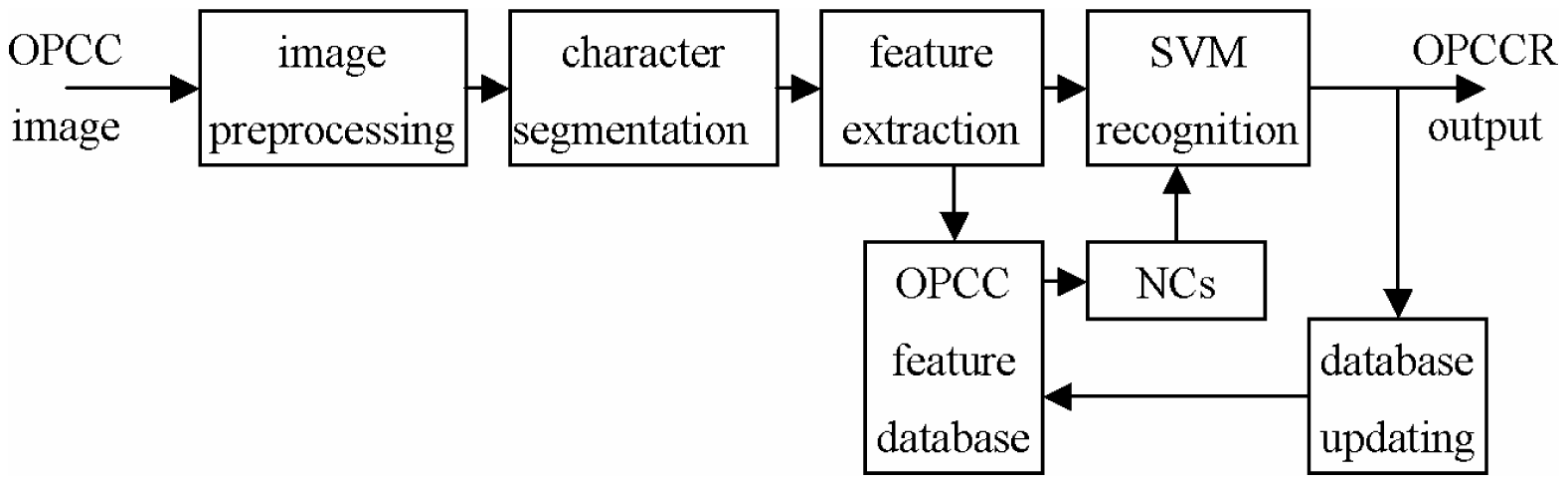
Overall diagram of the whole recognition system. The OPCC image is preprocessed to reduce the nonuniform illumination. Then it is segmented into individual character images. The features of individual character image are extracted and saved in OPCC feature database. From the OPCC feature database, the container of each pattern subspace is constructed. An unknown sample falling in a container is recognized by SVM using the NCs in the container. The OPCC feature database and the NCs in the container are updated by the output.

The overall diagram of the whole recognition system is shown in Figure 2. First, an optical printed Chinese character image is preprocessed to reduce the non uniform illumination. Next, the image is segmented to obtained individual unknown characters. Then, the features of an unknown character are extracted. From OPCC feature database, the neighbor classes of the unknown character are selected. Finally, the unknown character is recognized by SVM using the neighbor classes. Meanwhile, the OPCC feature database is updated by SVM using the output.

Some feature extraction methods can be used as reported in references [16]–[30]. However, these ways are either complicated, or time-consuming. For this reason, a simple probability of the stroke points (PSP) feature extraction can be adopted as the follows.

Let *b(x, y)* be the binary image of individual OPCC. Here, *x* = 0, 1, …, *H*−*1* and *y* = 0, 1, …, *W*−*1*, *H* is the height and *W* the width of the image of OPCC. In the binary image *b(x, y)*, a pixel with value 1 (one, in black) is a stroke point, and a pixel with value 0 (zero, in white) is a background point. The binary image *b(x, y)* is divided into *R*×*C* blocks. Here, *R* is the number of rows, and *C* the number of columns of the blocks. Then, the height h of a block is *h = H/R* and the width *w = W/C*. For each block, the PSP is calculated as a feature:
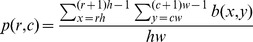
(19)


Thus, *D = R*×*C* features are extracted to form a *D* dimensional pattern *P^D^*, as described in Equation (1). The dimension number *i* of *p_i_* is calculated by:
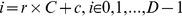
(20)


For each dimension *i*, the range of *p_i_* is divided into unit *u_ij_*, as described in Equation (2). The unit number *j* of *u_ij_* is calculated by Equation (3). Then pattern space *P^D^* is partitioned into *U^D^* subspaces, as described in Equation (4) and (5). Corresponding to each subspace {*u_ij_*}, a container *c_m_* is constructed. The number *m* is calculated according to Equation (5) and (6). The class numbers of the characters are stored into the corresponding container:
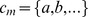
(21)


An example of a container and stored classes is shown as in figure 1, where container 4, i.e. container *c_4_* at (1, 1) contains classes 1, 2 and 3.
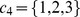
(22)


In the training procedure of NC-SVM, select a training sample from a class. Find the container of the sample according to Equation (5) and (6) and the neighbor classes of it in the container according to Equation (21). For example in Figure 1, select the training sample of class 2 in bald square and the training sample of class 3 in bald triangle. The two samples are in container 4 and their neighbor classes are class 1, 2 and 3. Then use the neighbor classes to train SVM.

In the classification procedure of NC-SVM, the container of an unknown sample is found according to Equation (5) and (6) and the neighbor classes of it in the container according to Equation (21). For example in Figure 1, the unknown sample in grey star locates in container 4. Its neighbor classes are class 1, 2 and 3. Then use the neighbor classes and SVM to classify the unknown sample.

## Experiments

To test our method of NC-SVM classification in OPCCR, some experiments have been done. First, the images of OPCCs were acquired and preprocessed. Then the extraction of the PSP features and the recognition of the OPCC were performed.

### 3.1 Image Acquisition and Processing

OPCCs with different font styles and sizes were printed on sheets of paper. The images of the sheets were acquired using digital camera. The images were enhanced using adaptive background correction to eliminate the non uniform illumination. The adaptive background correction is defined as:
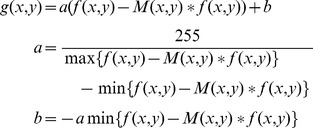
(23)


Where *M(x, y)* is a mean filter with size of *R*×*C*, *f(x, y)* is the acquired image, *g(x, y)* is the enhanced image. *R* and *C* are the row and column of the filter. They are predesigned according to the image size. In our experiments, *R* = 0.05*D*, *C* = 0.05*D*, *D* is the minimum of the row and the column of the image.

Since SVM classifier is suitable for the classification of dispersing samples, the distortion of the image is small and not rectified in our experiments.

**Figure 3:**
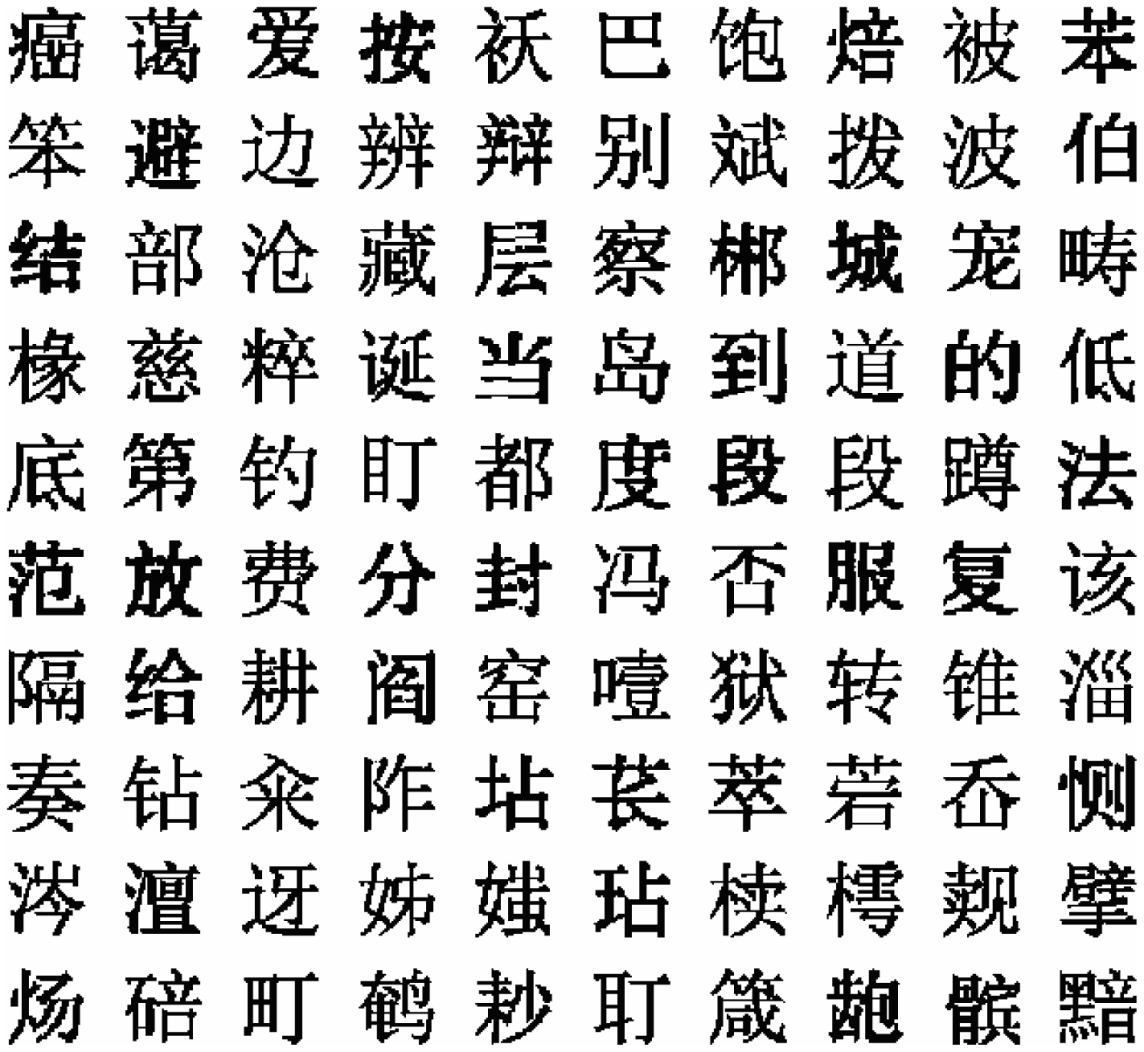
Some samples of OPCCs with font style SimSun. 100 individual OPCC binary images are presented. These images are preprocessed by adaptive background correction method to reduce the nonuniform illumination and normalized to 64×64.

Then they were segmented into binary images by threshold method. The binary images were cut into individual characters by projection method to obtain OPCCs. Some character examples are shown in figure 3. The OPCCs were stored in a Chinese character sample database for PSP feature extraction.

### 3.2 PSP Feature Extraction

Each of the preprocessed individual character images was divided into 16×16 blocks. In each block, the probability of the stroke points was calculated according to Equation (19). The probability was taken as the feature of the block. Thus, 16×16 features were obtained and a 16×16 probabilistic image was constructed for the character image. From the probabilistic image, the pattern of the character was formed according to Equation (1). The dimension of the pattern was 16×16. The patterns of the characters were stored in a Chinese character feature database for training and recognition.

### 3.3 Pattern Space Partitioning and Container Construction

In OPCCR, the dimension 16×16 of the pattern space is too big for subspace partitioning and container construction. On one hand, if each dimension is divided into 10 units, the pattern space will be partitioned into 10^16×16^ subspaces and 10^16×16^ containers need to be constructed. On the other hand, in practice, there are about 6,000 commonly used Chinese characters, hence, many subspaces would be empty and many subspaces would only contain one class. For this reason, we reduced the dimension 16×16 to 2×2 in the following way: The 16×16 probabilistic image was divided into 2×2 = 4 blocks. In each block, the probabilities were summed up according to Equation (19). Thus the pattern space was reduced to 4 dimensions. Each dimension was divided into 10 units and the pattern space was partitioned into 10^4^ = 10,000 subspaces. Correspondingly, 10,000 containers were constructed. The index of each container was calculated according to Equation (6). Then, the class numbers of the characters in a subspace were put into the corresponding container according to Equation (3) and (6).

### 3.4 Training of NC-SVM for OPCCR

In the feature database, for every font style and font size, each Chinese character contains 60 samples. Firstly, we selected randomly 30 samples as training samples. According to container construction method in Section 6.3, the container and the neighbor classes of each training sample are found. Then, SVM was used to determine the super-planes and the support vectors between the neighbor classes. The super-planes were stored in a Chinese character classifier database for OPCCR. The support vectors were stored in a Chinese character support vector database for updating training.

### 3.5 Recognition of OPCCs Using NC-SVM for Selected Similar Characters

In this experiment, we firstly selected manually 368 similar characters from GB2312-80 standard as test objectives, which were sorted into 236 groups according to their character structure. We selected the rest 30 samples for each character as the test samples. According to Section 6.3, found the container and the neighbor classes of each testing sample. The radial basis function (RBF) is selected as kernel function for this experiment; penalty *C* and *γ* of RBF are 100 and 0.00390625 for all NC-SVM classifiers [31]. Then, the super-planes between the neighbor classes were selected to recognize the test sample. Meanwhile, the test sample together with the support vectors between the neighbor classes were used to updating training.

First, the test samples with same font style SimSun and different font size 12, 18, 26 and 36 are selected. Table 1 shows the classification rate of 236 groups similar characters.

**Table 1:** IR of different font size, the font style is SimSun.

Font Size	Correct Numbers	Error Numbers	IR (%)
12	3435	245	96.67
18	3582	98	97.34
26	3600	80	97.83
36	3637	43	98.83

Then we chose these similar groups with 4 font styles: SimSun, SimHei, KaiTi_GB2312, and FangSong_GB2312 with the same font size 18 from the Chinese character sample database. The styles and the font size are normally used in books and document files. Table 2 shows classification rates for 236 groups similar characters.

**Table 2:** IR of different font style, the font size is 18.

Font Style	Correct	Error	IR (%)
SimSun	3551	129	96.49
SimHei	3551	129	96.49
KaiTi_GB2312	3545	135	96.33
FangSong_GB2312	3527	153	95.84

### 3.6 Comparison with Other Methods

To illustrate the effectiveness of IR and the computation reduction of our method, we present a comparison process with SVM-KNN [6], GsvmLBDknn [7], SVM-NNC [8], LASVM [9], and MQDF2 [15].

We focus on the trade off between the OPCCR identification rate (IR) and computation consuming (CC). Two experiments are given in this section. In the first experiment, OPCCs dataset is used. In the dataset, all Chinese characters of GB2312-80 are taken into this simulation. Each character of font type SimSun with font size 18 contains 60 samples, 30 of them are chosen randomly as training samples in the learning and training process, 30 of the left are chosen as test samples in the testing process. The PSP features of each sample are extracted by Equation (19). The NCs of all pattern features are constructed. According to Reference [6], the *Ksl* neighbors are 800, and the *K* neighbors based on *Ksl* are 80. The optional *Kl* of a query’s neighbors in Reference [7] is 200. In the method of Reference [9], the parameter *k* of nearest neighbors is selected as 20. To verify the generalization of our method, three kernel functions of SVM commonly utilized are presented, i.e., the linear kernel, the third order polynomial kernel and RBF kernel. According to the tools of LIBSVM [31], penalty parameter *C* is selected as 100 and *γ*of RBF is 0.00390625 for all classifiers. In MQDF2, eigenvalues of covariance matrix are sorted in descending order. The value of *k* which indicates the number of eigenvalues and eigenvectors should be reserved is 20, and *h^2^* is chosen as the average of *λ_21_* for all classifiers. The simulation results, including IR and CC for each query sample averagely (seconds per sample), are shown in Table 3, Table 4, and Table 5. The methods in experiment are briefly denoted as: M1 for SVM-KNN, M2 for GsvmLBDknn, M3 for SVM-NNC, M4 for LASVM, M5 for NC-SVM, and M6 for MQDF2.

**Table 3:** The IR and CC for OPCCs dataset.

method	M1	M2	M3	M4	M5	M6
IR (%)	97.79	97.57	96.41	97.65	97.81	97.81
CC (s)	2.132	2.046	1.957	1.485	0.385	0.334

The linear function for M1– M5.

**Table 4:** The IR and CC for OPCCs dataset.

method	M1	M2	M3	M4	M5	M6
IR (%)	98.49	98.34	98.40	98.31	98.48	97.12
CC(s)	2.278	2.134	2.049	1.957	0.393	0.446

The kernel function is third order polynomial kernel function for M1– M5.

**Table 5:** The IR and CC for OPCCs dataset.

method	M1	M2	M3	M4	M5	M6
IR (%)	98.65	98.17	98.22	98.39	98.70	96.91
CC(s)	2.276	2.046	1.971	2.123	0.368	0.438

RBF is chosen as kernel function for SVM based methods M1– M5.

In the second experiment, the six methods, M1 to M6 are performed on the MNIST dataset. The MNIST database of handwritten digits contains 60,000 training examples, and 10,000 testing examples. The original images in the dataset are normalized to the size of 64×64 pixels. The procedures of the digit classification in the six methods are the same as the OPCCs dataset but the parameters selected are different from the OPCCs dataset. Parameter *C* and *γ* of RBF are 120 and 0.001. The experiment results are listed in Table 6, Table 7, and Table 8.

**Table 6:** The IR and CC for MNIST dataset.

method	M1	M2	M3	M4	M5	M6
IR (%)	96.52	96.31	96.34	96.48	96.51	96.34
CC(s)	0.826	0.760	0.797	0.754	0.395	0.349

The linear function is applied in M1– M5.

**Table 7:** The IR and CC for MNIST dataset.

method	M1	M2	M3	M4	M5	M6
IR (%)	98.54	98.46	98.51	98.47	98.60	98.36
CC(s)	0.989	0.869	0.952	0.946	0.406	0.389

The kernel function utilized in SVM is third order polynomial kernel function for method M1– M5.

**Table 8:** The IR and CC for MINST dataset.

method	M1	M2	M3	M4	M5	M6
IR (%)	98.69	98.65	98.38	98.59	98.72	96.89
CC(s)	1.059	0.959	0.909	0.986	0.419	0.385

RBF is chosen as kernel function for SVM based methods M1– M5.

From these tables we can see that the IRs of the methods are not significantly different. The CCs of the last two methods M5 and M6 are much smaller than those of the first four methods M1 to M4. Compared with M6 (MQDF2), M5 (NC-SVM) has slightly bigger computer consumption. The main distinction of NC-SVM is that its computation consumption decreases observably than other four locality based SVM methods. For the SVM-KNN method, to pick the *k* nearest neighbor samples we should compute distance of the query to all training samples. In our OPCCs database, the class numbers are 6773 and the training samples are enormous, thus the computation consumption for the *k* nearest neighbor samples increases obviously. The selection of *k* local neighbors is also sensitive to IR. The IR falls with small local neighbor samples because some local classes nearby query sample are excluded from local nearest neighbors. The GsvmLBDknn takes a good recognition result. When the global SVM fails, the local SVM is adopted to learn a query-dependent boundary-driven metric by which the *K* nearest neighbors are found. Then the KNN classifier is formed to classify the query sample. Considering the OPCCs database with large classes, the global SVM process expends much more computing time. So the computation consumption increases rapidly on account of the hybrid of global SVM and local SVM based KNN classifier. The SVM-NNC firstly utilizes global SVM classifiers to identify the query sample as well. This global SVM classifiers process spends apparently a plenty of computing time that results in increasing of computation consumption. The GsvmLBDknn and SVM-NNC are appropriate for pattern recognition with small class numbers, since the computation time is affected indistinctively by global SVM process. LASVM is also applicable for recognition with small number of classes. For the database containing large classes, while the global one-versus-all SVM process works if the labels of all *k* neighbors of query sample are different, the computation of this global SVM will increase quickly as well.

The NC-SVM considers the neighbor classes of a query sample, not the *k* nearest neighbor samples. Thus, all classifiers of its local neighbors are involved the container corresponding to the query sample completely; the IR is maintained satisfactorily. Meanwhile, the neighbor classes included in each container is much less than the whole class number of OPCCs which is illustrated in Section 3. Thus, the computation consumption of recognition decreases dramatically comparing with other SVM based methods. The simulation result illustrates the effectiveness of computation reduction of NC-SVM.

## Discussion and Conclusion

SVM is an optimal classifier. In many cases, using SVM classifier can achieve high IR. However, in the case of multi classes, SVM is computational time-consuming. Thus, in our OPCCR, we propose an NC-SVM method to reduce the computational time of SVM. The NC-SVM method has similar identification rate (IR) to those of the five methods: SVM-KNN, GsvmLBDknn, SVM-NNC, LASVM and MQDF2. The computational time-consuming (CC) is much less than those of the first four methods, but slightly greater than that of the method MQDF2.

In training stage, only the training samples in the neighbor classes (NCs) but not all the training samples are used to train the SVM classifier. In the classification stage, only the neighbor classes but not all the classes are selected to recognize the unknown sample. The number of NCs is less than that of all Chinese character classes, thus the training time and classification time are much reduced.

In the pattern space of OPCCR, the samples in the neighbor classes of an unknown sample are close to the unknown sample, but the other samples of the other classes are far away the unknown sample. Hence the other samples not being used in the training stage and classification stage would not affect the identification rate. Thus, the IR of NC-SVM is not significantly different from those of the five methods.

In NC-SVM, the pattern space partitioning would affect the IR and CC of OPCCR. The bigger the subspace, the larger the NCs are and the bigger the IR is but the bigger the CC is. Vice versa, the smaller the NCs are and the smaller the IR is but smaller the CC is. The relationship between the partitioning and IR/CC will be studied in the future.
