# Correction: A Method of Neighbor Classes Based SVM Classification for Optical Printed Chinese Character Recognition

**DOI:** 10.1371/annotation/f089122e-03d9-4715-9d92-c284f8d8b416

**Published:** 2013-05-17

**Authors:** Jie Zhang, Xiaohong Wu, Yanmei Yu, Daisheng Luo

 In our publication in PLOS ONE, we failed to cite a previous conference publication related to this work, the authors apologize for this oversight and would like to include a citation to our previous article below:

Zhang J, Wu X, Yu Y, Luo D (2012) A Novel Method of Feature Extraction and Classification for OPCCR. Computer, Consumer and Control (IS3C), 2012 International Symposium on. IEEE: 717-720.

The aim of both papers is to reduce the computation load of the SVM method when it is utilized in OPCCR. Therefore the same Equation, which can be found as Equation (1) in Section II of the conference paper and Equation (19) in Section 5 of the PLOS ONE article, is adopted to calculate the regional probability distribution of the stroke pixels as the feature vector of a Chinese character image . In addition, two same concepts, i.e. pattern space partitioning and container, are introduced, which can be found in Section III of the conference article and Section 2 of the PLOS ONE article.

However, there are a number of differences between the two publications:

1) In the pattern space division process, the construction methods of pattern subspace are different.

2) The construction methods of the container, which is introduced to save the neighbor character classes with a pattern subspace, are different in the two studies.

3) In the PLOS ONE article, an overall diagram of the recognition system is proposed and how this system operates is explained in section 5. This was not reported in the conference publication.

4) The PLOS ONE article includes a previously unreported theoretical analysis for computation complexity reduction of NC-SVM, both in the training and classifying processes in section 3 and 4. The experiments analysis for computation complexity about NC-SVM compared with other SVM based methods and MQDF method is also provided in section 6.6 'Comparison with other methods.

Because of the similarity of the research question between the two articles, there are three similar fragments in their abstracts. However, these fragments are only common descriptions, and the NC-SVM proposed in the PLOS ONE article is different compared with the LNN-SVM in the conference publication. 

